# Redox‐Polymer‐Based High‐Current‐Density Gas‐Diffusion H_2_‐Oxidation Bioanode Using [FeFe] Hydrogenase from *Desulfovibrio desulfuricans* in a Membrane‐free Biofuel Cell

**DOI:** 10.1002/anie.202006824

**Published:** 2020-07-21

**Authors:** Julian Szczesny, James A. Birrell, Felipe Conzuelo, Wolfgang Lubitz, Adrian Ruff, Wolfgang Schuhmann

**Affiliations:** ^1^ Analytical Chemistry—Center for Electrochemical Sciences (CES) Faculty of Chemistry and Biochemistry Ruhr University Bochum Universitätsstr. 150 44780 Bochum Germany; ^2^ Max Planck Institute for Chemical Energy Conversion Stiftstrasse 34–36 45470 Mülheim an der Ruhr Germany; ^3^ Present address: PPG (Deutschland) Business Support GmbH, PPG Packaging Coatings Erlenbrunnenstr. 20 72411 Bodelshausen Germany

**Keywords:** biofuel cells, gas diffusion electrodes, hydrogenases, molecular hydrogen, redox polymers

## Abstract

The incorporation of highly active but also highly sensitive catalysts (e.g. the [FeFe] hydrogenase from Desulfovibrio desulfuricans) in biofuel cells is still one of the major challenges in sustainable energy conversion. We report the fabrication of a dual‐gas diffusion electrode H_2_/O_2_ biofuel cell equipped with a [FeFe] hydrogenase/redox polymer‐based high‐current‐density H_2_‐oxidation bioanode. The bioanodes show benchmark current densities of around 14 mA cm^−2^ and the corresponding fuel cell tests exhibit a benchmark for a hydrogenase/redox polymer‐based biofuel cell with outstanding power densities of 5.4 mW cm^−2^ at 0.7 V cell voltage. Furthermore, the highly sensitive [FeFe] hydrogenase is protected against oxygen damage by the redox polymer and can function under 5 % O_2_.

## Graphical abstract

**A highly active** but extremely O_2_‐sensitive [FeFe] hydrogenase that acts as a H_2_‐oxidation catalyst in a H_2_/O_2_ biofuel cell is integrated in a shielded bioanode and a membrane‐free H_2_/O_2_ biofuel cell. The enzyme was wired to gas diffusion electrodes by means of a viologen‐modified redox polymer matrix. Outstanding current densities of up to 14 mA cm^−2^ and power outputs of 5.5 mW cm^−2^ in a membrane‐free device were achieved.

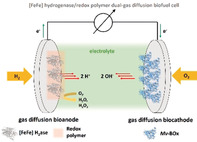

In the transition from fossil fuels to a sustainable energy economy, a sustainable solution to store excess electrical energy is to generate H_2_ by water electrolysis.^1^, ^2^, ^3^, ^4^ H_2_ can then be oxidized as a fuel on demand at an electrode modified with an appropriate electrocatalyst, while O_2_ is reduced to water at a suitable cathode.^3^ The most commonly applied electrocatalysts in H_2_/O_2_ fuel cells are based on scarce and costly noble metals like Pt and Ir.^1^ Evidently, for a sustainable and economic process, electrocatalysts based on abundant materials are highly desired.

Nature has evolved highly efficient biocatalysts, hydrogenases, for the reversible conversion of H_2_, using active centers consisting solely of earth‐abundant metals, namely Ni and Fe,^5^ and with a catalytic performance similar to that of Pt.^4^, ^5^, ^6^, ^7^ Hydrogenases have already been employed in the anode of H_2_/O_2_ biofuel cells,^7^, ^8^, ^9^, ^10^ typically in combination with multi‐copper oxidase (bilirubin oxidase or laccase)‐based O_2_‐reducing biocathodes.^11^ The high activity of hydrogenases correlates with high sensitivity towards O_2_ as well as high‐potential inactivation, which hampers their integration in potentially useful devices. Hence, such devices are restricted to the lab scale.^7^, ^8^, ^9^, ^12^, ^13^ In particular, [FeFe] hydrogenases are among the most active biocatalysts for H_2_‐oxidation; however, they suffer from extreme and irreversible O_2_ deactivation, rapid inactivation at high potentials, and degradation under light irradiation.^5^, ^7^, ^14^ While the less sensitive [NiFe] and [NiFeSe] hydrogenases have been integrated in high‐performance dual‐gas diffusion electrodes^15^, ^16^ as well as gas diffusion biofuel cells,^17^, ^18^, ^19^, ^20^ the use of [FeFe] hydrogenases in such devices has not—to the best of our knowledge—been described so far and applications are often limited to glovebox conditions.^14^, ^21^, ^22^

The use of low‐potential viologen‐modified redox polymers as immobilization, wiring, and protection matrix^23^, ^24^, ^25^ ensured the use of various hydrogenases in conventional^22^, ^23^ and membrane‐free dual‐gas diffusion H_2_/O_2_ biofuel cells.^20^, ^26^ Even the highly sensitive [FeFe] hydrogenase could be integrated in a conventional H_2_/O_2_ biofuel cell in combination with an O_2_‐reducing bilirubin oxidase biocathode.^22^ However, the system was limited by mass transport and the power output was similar to fuel cells based on [NiFe]^23^ and [NiFeSe]^26^ hydrogenases. Thus, immobilization of the highly active [FeFe] hydrogenase into gas diffusion layers is greatly desired.^12^, ^15^, ^27^ Stimulated by our previous findings,^20^, ^26^ we exploited the possibility to construct a redox polymer/[FeFe] hydrogenase high‐current‐density gas diffusion bioanode for H_2_‐oxidation that can be introduced into a membrane‐free biofuel cell. As the active H_2_‐oxidation catalyst we selected the [FeFe] hydrogenase from *Desulfovibrio desulfuricans* (DdHydAB), which is one of the most active hydrogenases for H_2_‐oxidation with turnover frequencies of up to 63 000 s^−1^.^14^, ^21^

As the electrode material, carbon‐cloth‐based gas diffusion layers were used for the immobilization of polymer/hydrogenase layers and the construction of high‐current‐density H_2_‐oxidation bioanodes as shown earlier for other types of hydrogenases.^20^, ^26^

For the electrical wiring of DdHydAB, the redox polymers P(N_3_MA‐BA‐GMA)‐vio (poly(3‐azido‐propyl methacrylate‐*co*‐butyl acrylate‐*co*‐glycidyl methacrylate)‐viologen) and P(GMA‐BA‐PEGMA)‐vio (poly(glycidyl methacrylate‐*co*‐butyl acrylate‐*co*‐poly(ethylene glycol)methacrylate)‐ viologen) were integrated in a double‐layer configuration (Figure 1) analogous to systems based on [NiFe] and [NiFeSe] hydrogenases that were described previously.^20^, ^25^ The less hydrophilic P(GMA‐BA‐PEGMA)‐vio (lower viologen content, <65 mol %, compared to P(N_3_MA‐BA‐GMA)‐vio, 71 mol %) is used as an adhesion layer between the active P(N_3_MA‐BA‐GMA)‐vio/hydrogenase layer (green part in Figure 1) and the rather hydrophobic carbon cloth.^20^ Both polymers are modified with the same viologen species with the redox potential of ≈−0.3 V vs. SHE,^20^ which is approximately 0.11 V more positive than the potential of the H_2_/H^+^ couple at neutral pH and at 298.15 K (−0.414 V vs. SHE). This allows for a high driving force for H_2_‐oxidation but is still low enough to ensure a high open circuit voltage (OCV) in a corresponding biofuel cell that depends on the potential gap between bioanode and biocathode.^30^ We want to emphasize that redox polymers additionally act as pseudo‐capacitive elements which further increase the OCV due to charging of the immobilized polymer matrix leading to a shift in open circuit potential under turnover conditions (see Note S1 in the Supporting Information and refs. 31, 32).

**Figure 1:**
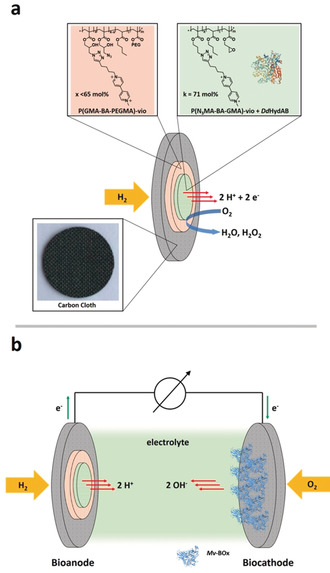
a) Scheme of the proposed multilayer gas diffusion electrode modified with the DdHydAB (1HFE^28^) hydrogenase wired through the viologen‐modified polymers P(GMA‐BA‐PEGMA)‐vio and P(N_3_MA‐BA‐GMA)‐vio. b) Schematic of the dual gas diffusion membrane‐free H_2_/O_2_ powered biofuel cell equipped with the bioanode depicted in (a) and an O_2_‐reducing bilirubin oxidase (6IQZ^29^) based biocathode operated in a direct electron transfer regime. Not drawn to scale.

The carbon‐cloth‐based electrodes were modified in a sequential drop‐cast process. First the (P(GMA‐BA‐PEGMA)‐vio) adhesion layer was deposited and dried on the electrodes followed by immobilization of the catalytically active layer (P(N_3_MA‐BA‐GMA)‐vio/*Dd*HydAB). The double‐layer electrodes will be designated as P(GMA‐BA‐PEGMA)‐vio//P(N_3_MA‐BA‐GMA)‐vio/*Dd*HydAB in the following. The electrodes were prepared under glovebox conditions. For measurements, the electrodes were transferred under Ar atmosphere using standard Schlenk techniques and mounted into the gas diffusion electrochemical cell^20^ that was installed in a conventional fumehood.

Cyclic voltammograms (Figure 2 a) recorded with P(GMA‐BA‐PEGMA)‐vio//P(N_3_MA‐BA‐GMA)‐vio/DdHydAB electrodes under turnover conditions (H_2_ gas diffusion mode, red curve) show pronounced catalytic oxidation waves with absolute steady state currents of up to 2.0 mA at +0.2 V vs. SHE, which accounts for a current density of ≈16 mA cm^−2^ (referenced to the modified surface area on the carbon cloth substrate, diameter of polymer spot ≈4 mm). The half‐wave potential of the catalytic wave (−0.16 V vs. SHE) is slightly above the midpoint potential of the polymer‐bound viologen species (Figure 2 a, black curves, −0.3 V); however, the onset potential of the H_2_‐oxidation wave matches the redox potential of the polymer‐bound viologen units confirming mediated electron transfer, which is crucial for an effective protection of the sensitive enzyme.^23^ The shift of the half‐wave potential to more positive values as compared to the midpoint potential of the mediator might be due to *iR* drop at these rather high current values. This is corroborated by the large peak potential separation of ≈130 mV in the voltammograms recorded under non‐turnover conditions. High potential inactivation at >0 V vs. SHE is absent, showing that the polymer indeed acts as a potential shield (Nernst buffer*)* 23 for this sensitive protein.

**Figure 2:**
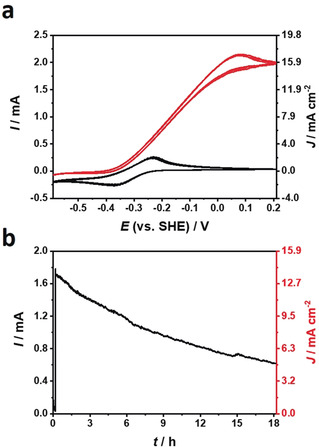
Electrochemical characterization of the redox polymer/DdHydAB‐based bioanode. a) Cyclic voltammograms (5 mV s^−1^) of a P(GMA‐BA‐PEGMA)‐vio//P(N_3_MA‐BA‐GMA)‐vio/DdHydAB gas‐diffusion bioanode in the presence of H_2_ (red traces, three consecutive CVs) and Ar (black traces, three consecutive CVs). B) Chronoamperometry of a P(GMA‐BA‐PEGMA)‐vio//P(N_3_MA‐BA‐GMA)‐vio/DdHydAB bioanode at an applied potential of +0.16 V vs. SHE and in H_2_ gas diffusion mode. Working electrolyte: 0.1 m phosphate buffer (pH 7.4); nominal hydrogenase loading: 39.8 nmol cm^−2^, total polymer loading: 1.8 mg cm^−2^.

Average H_2_ steady‐state oxidation currents were calculated to be 1.8±0.3 mA/14.1±2.1 mA cm^−2^ (*n*=3; Table S1). The values exceed current densities obtained in direct electron transfer, with the enzyme wired to graphite electrodes (350–450 μA cm^−2^),^14^, ^21^ demonstrating the advantage of the 3D polymer matrix which ensures wiring of a large amount of enzyme independent of its orientation and location on the electrode surface as well as the enhanced mass transport ensured by the gas diffusion layer.

The operational stability of the P(GMA‐BA‐PEGMA)‐vio//P(N_3_MA‐BA‐GMA)‐vio/DdHydAB electrode was evaluated by chronoamperometry showing a current loss of ≈50 % of the initial current after 11 h (Figure 2 b), while after 18 h of operation the current dropped to 35 % of the initial value. In comparison, a gas diffusion electrode based on a less sensitive [NiFe] hydrogenase wired with the same redox polymer double‐layer architecture shows a 50 % drop after ≈13 h.^20^ The protection capability of the polymer towards O_2_ damage was analyzed by means of chronoamperometry, while varying gas mixtures were purged through the electrolyte solution but with a constant supply of H_2_ from the backside of the gas diffusion electrode (Figure 3 a). When O_2_ (5 %/95 % Ar) enters the electrolyte, the current decreases since some of the electrons liberated from H_2_‐oxidation are used by the redox polymer matrix to reduce incoming O_2_ molecules.^23^, ^24^

**Figure 3:**
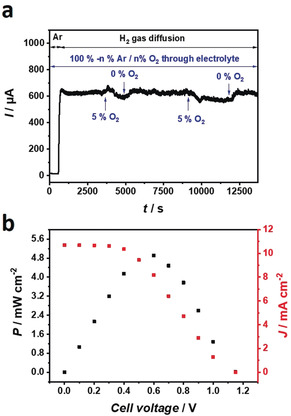
Characterization of bioanode stability against O_2_ (a) and performance of the H_2_/O_2_ biofuel cell equipped with a P(GMA‐BA‐PEGMA)‐vio//P(N_3_MA‐BA‐GMA)‐vio/DdHydAB bioanode (polymer loading: 1.8 mg cm^−2^) coupled to a Mv‐BOx‐based biocathode in 0.1 m phosphate buffer, pH 7.4 (b). a) Chronoamperometry at an applied potential of +0.16 V vs. SHE in H_2_ gas diffusion mode at changing gas mixtures (Ar (100 %) or Ar/O_2_ (95 %/5 %)) purged through the electrolyte; nominal enzyme loading: 19.9 nmol cm^−2^. B) Power curve of the fully assembled membrane‐free dual‐gas diffusion H_2_/O_2_ biofuel cell showing power (left ordinate, black) and current densities (right ordinate, red); nominal enzyme loading: 39.8 nmol cm^−2^.

After the gas feed was changed back to Ar, the initial currents were restored, indicating efficient protection of the highly sensitive DdHydAB by the polymer matrix even on the porous and rather rough carbon‐cloth‐based electrode (note that protection is most efficient for homogeneous and smooth polymer/enzyme layers^24^, ^33^). This result is in line with our previous findings concerning gas diffusion electrodes^20^, ^26^ and for conventional flat glassy carbon electrodes.^22^, ^23^, ^25^

To evaluate the performance of the [FeFe]/polymer‐based bioanode in a biofuel cell, we combined the H_2_‐oxidation electrode with a O_2_‐reducing bilirubin oxidase (from *Myrothecium verrucaria*, Mv‐BOx) gas diffusion biocathode (see the Supporting information for preparation conditions). The maximum power density of the dual‐gas diffusion biofuel cell under anode limiting conditions (Figure S1) was 5.4 mW cm^−2^ at 0.7 V, which sets a new benchmark for a biofuel cell equipped with a polymer/hydrogenase‐based bioanode (Table S1). The OCV was 1.13 V (recorded after a constant voltage was obtained at open circuit, reached within 3 min) and is only slightly lower than the theoretical value of 1.23 V. Moreover, the value is similar to that of [NiFe]‐ and [NiFeSe]‐based dual‐gas diffusion biofuel cells operated in a mediated electron transfer regime^20^ and in a direct electron transfer regime.^17^, ^18^ Evidently, the pseudo‐capacitive effect of the polymer matrix^31^, ^32^ compensates for the fact that the redox potential of the viologen is slightly higher than that of the hydrogenase. Cyclic voltammograms recorded with the bioanode after biofuel cell tests show identical current values under turnover and non‐turnover conditions (Figure S2 a). The operational stability of the membrane‐free device was tested at a constant load of 0.7 V. The power output decreases after 16 h to 60 % of the initial value under continuous operation. This demonstrates the protection capability of the polymer matrix for the sensitive [FeFe] hydrogenase even in a membrane‐free device and even outperforms the previously reported polymer/[NiFe] gas diffusion system (50 % after 7 h) measured under identical conditions.^20^ In contrast, when a H_2_‐oxidation catalyst with a higher O_2_ stability is used as the active material, such as a [NiFeSe] variant,^34^ the operational stability of the device is drastically enhanced (75 % of the initial current remains after 10 h of continuous operation).^26^ Hence, we can conclude that the overall stability is mainly based on the intrinsic properties of the enzyme.

Cyclic voltammograms recorded with the redox polymer/DdHydAB bioanode and the O_2_ reducing biocathode before and after the long‐term measurement show that the bioanode (Figure S2 a) as well as the biocathode (Figure S1) exhibit a substantial drop in performance. The stability of the anode remains surprisingly high considering the operating conditions (anode limiting conditions in a membrane‐free device, operated in a regular fumehood) and the high sensitivity of DdHydAB to immediate O_2_ damage.

The maximum current densities (*J*_max_) for H_2_‐oxidation and the corresponding maximum power density (*P*_max_) in the dual‐gas diffusion biofuel cell obtained with the DdHydAB [FeFe] hydrogenase are significantly higher than those previously reported for the [NiFe] hydrogenase (*J*_max_=7.9 mA cm^−2^; *P*_max_=3.6 mW cm^−2^ at 0.7 V),^20^ the wild‐type [NiFeSe] hydrogenase (*J*_max_=5.3 mA cm^−2^),^20^ and a [NiFeSe] hydrogenase variant^34^ with enhanced O_2_ resistance (*J*_max_=6.3 mA cm^−2^; *P*_max_=4.4 mW cm^−2^ at 0.7 V)^26^ (Table S1).

However, when analyzing the solution activities for the individual hydrogenases (Table S1) even higher current densities could be expected for the DdHydAB [FeFe] hydrogenase. Because of the enhanced mass transport under gas diffusion conditions, limitations due to substrate transport can be ruled out. Consequently, electron transport within the redox polymer matrix and/or limitations due to local pH changes because of the massive generation of protons at the electrode–gas–electrolyte interface must be rate limiting. Moreover, the delicate interplay between the individual components (catalyst, redox polymer, and electrode surface) seems to be crucial for the overall activity of the H_2_‐oxidation anode. This becomes clear when comparing the enzyme loading for different gas diffusion electrodes (Table S1). In particular, the structurally modified variant of the [NiFeSe] hydrogenase shows outstanding performance even at low catalyst loadings, which was attributed to the enhanced O_2_ resistance of this catalyst (all enzymes are in an active state) and a more favored interaction between the polymer matrix/enzyme/electrode interface because of the intrinsic properties of this structurally modified variant, polymer and electrode surface.^26^ However, the presented results show that even highly sensitive catalysts such as DdHydAB [FeFe] hydrogenase can be successfully incorporated into high‐current‐density gas diffusion H_2_‐oxidation electrodes and can be used as bioanode in a membrane‐free H_2_/O_2_ powered biofuel cell with outstanding performance that sets a benchmark with respect to current and power densities under anode‐limiting conditions. Moreover, the protection capability of the polymer matrix allows for operation even avoiding complex glovebox systems which is crucial for the transformation of this system to potentially technologically relevant systems.

## Conflict of interest

The authors declare no conflict of interest.

## Supporting information

As a service to our authors and readers, this journal provides supporting information supplied by the authors. Such materials are peer reviewed and may be re‐organized for online delivery, but are not copy‐edited or typeset. Technical support issues arising from supporting information (other than missing files) should be addressed to the authors.

Supplementary
