# Supplementary material for: Redox‐Polymer‐Based High‐Current‐Density Gas‐Diffusion H2‐Oxidation Bioanode Using [FeFe] Hydrogenase from Desulfovibrio desulfuricans in a Membrane‐free Biofuel Cell
Source: Angew Chem Int Ed Engl. 2020 Jul 21;59(38):16506–10. doi: 10.1002/anie.202006824 (PMC7540381; doi:10.1002/anie.202006824)
Supplement: Supplementary file 1 — Supplementary [file ANIE-59-16506-s001.pdf]

Angewandte Chemie
Eine Zeitschrift der Gesellschaft Deutscher Chemiker

Supporting Information

Redox-Polymer-Based High-Current-Density Gas-Diffusion H_2-Oxidation Bioanode Using [FeFe] Hydrogenase from *Desulfovibrio desulfuricans* in a Membrane-free Biofuel Cell

Julian Szczesny, James A. Birrell, Felipe Conzuelo, Wolfgang Lubitz, Adrian Ruff,* and Wolfgang Schuhmann**

anie_202006824_sm_miscellaneous_information.pdf

Experimental Section

Materials and chemicals

All chemicals were purchased from Sigma-Aldrich, Merck, Acros Organics, abcr, Alfa Aesar, J. T. Baker Fisher Chemicals or VWR and were of reagent or higher grade and were used as received. All aqueous and buffer solutions were prepared with deionized water from a Millipore system. The synthesis of the redox polymers P(N_3MA-BA-GMA)-vio (poly(3-azido-propyl methacrylate-*co*-butyl acrylate-*co*-glycidyl methacrylate)-viologen) and P(GMA-BA-PEGMA)-vio (poly(glycidyl methacrylate-*co*-butyl acrylate-*co*-poly(ethylene glycol)methacrylate)-viologen) was described elsewhere in refs. [1] and [2].

Enzymes

[FeFe] hydrogenase from *Desulfovibrio desulfuricans* was prepared as described in[3]. The enzyme was stored as a 2 mM solution in 25 mM Tris-HCl buffer, 25 mM KCl, pH 8 at -80 °C. Bilirubin oxidase from *Myrothecium verrucaria* was purchased from Sigma-Aldrich and was stored at -20 °C. For electrode preparation it was dissolved in a 0.1 M potassium phosphate buffer (PB) pH 7.4 in a concentration of 15 mg ml^{-1}.

Electrochemical Experiments

All electrochemical experiments were conducted at room temperature under an inert gas atmosphere (Ar or H_2/Ar mixtures) in an electrochemical gas diffusion cell (see ref. [2] for details) mounted in a conventional fume hood. A standard three-electrode setup was used to investigate individual electrodes. For fuel cell performance tests, a two-electrode configuration was used. As working electrodes, carbon cloth based gas diffusion layers ((MTI, Carbon Foam Sheet, Porous C, 0.454 mm thick, ≈10 mL cm^{-2} s^{-1}, porosity ≈31µm; note that one side of the carbon cloth is coated with a Nafion/Teflon-based microporous film (50 µm), carbon content 5 mg cm^{-2}, EQ-bcgdl-1400S-LD, this side was denoted as the microporous side/layer, the non-modified side was denoted as macroporous side/layer) were used. As the counter electrode in the three-electrode setup a Pt-wire with a diameter of 1 mm was used. As reference electrodes, a commercial (Methrom) Ag/AgCl/3 M KCl system was used. All measured potentials are rescaled vs. SHE according to $E_{SHE} = E_{Ag/AgCl/3\ M\ KCl} + 210$ mV. All measurements were performed with a Reference 600 (Gamry Instruments) or an Interface 1000 (Gamry Instruments) potentiostat. Scan rates for cyclic voltammograms and applied potentials for chronoamperograms as well as the used gas mixtures are noted in the

corresponding figure captions. Phosphate buffer (PB, 0.1 M, pH 7.4) was used as the working electrolyte for all experiments. Mass flow controllers were used to control the composition of the gas feed. For biofuel cell characterization, steady state currents from multi-step chronoamperometry were used to calculate power values for the corresponding power curves to minimize the contribution from capacitive currents. In gas-diffusion mode a backpressure of the corresponding gas was applied to the gas diffusion layer, while the electrolyte was continuously flushed with Ar (note that because of pressure equilibration, the cell was not fully closed).

Electrode preparation

Bioanode. For preparation of the bioanode, the macroporous side of the carbon cloth electrodes were used to prevent direct electron transfer. In a first step, 20 µL of an aqueous P(GMA-BA-PEGMA)-vio solution (7.5 mg mL^{-1}) was drop cast onto the electrode over an area of approximately 4 mm as an adhesion layer and dried for 12 h at room temperature. In a second step, the electrodes were transferred to an O_2 free glove box and 10 µL of an aqueous P(N_3MA-BA-GMA)-vio solution (8 mg mL^{-1}) were mixed with 2.5 µL of the *Dd*HydAB hydrogenase stock solution (2 mM in 25 mM Tris-HCl buffer, 25 mM KCl, pH 8) and drop coated on top of the adhesion layer and dried for 12 h at room temperature (for O_2 stability measurements only 1.5 µL of the *Dd*HydAB solution were used). The electrodes reveal nominal hydrogenase loadings of 39.8 nmol cm^{-2} (O_2 stability experiments: 19.9 nmol cm^{-2}). The nominal polymer loadings of P(GMA-BA-PEGMA)-vio and of P(N_3MA-BA-GMA)-vio were 150 µg/1.2 mg cm^{-2} and 80 µg/0.6 mg cm^{-2} (total polymer loading = 1.8 mg cm^{-2}), respectively, for all bioanodes. This polymer/enzyme ratio showed the best performance (several polymer/enzyme ratios were tested, data not shown) and were used for all measurements reported in this work (see text and corresponding figure captions). For electrochemical measurements in a conventional fume hood, the electrodes were transferred under an argon atmosphere.

Biocathode. For preparation of the biocathode, the microporous side of the carbon cloth electrodes was used (geometrical surface area: 3.5 cm^2, note that the actual surface area is much higher due to the porous nature of the electrode material) to ensure a high catalyst loading and productive wiring in a direct electron transfer regime. The high surface area of the biocathode compared to the bioanode ensures anode limiting conditions. First, the electrode was washed with ethanol and water.

Subsequently, the surface of the electrode was modified with 2-aminobenzoic acid in an electrochemical grafting process in an aqueous solution of 0.1 M KCl and 5 mM 2-aminobenzoic acid. A potential of 800 mV vs. Ag/AgCl 3 M KCl was applied for 60 s as described in ref. [4]. After the grafting process, the electrode was washed with water and 80 µl of the *Mv*-BOx solution (15 mg mL^{-1} in 0.1 M PB pH 7.4) were drop cast on the chemically modified electrode. The electrode was dried for 1.5 h at room temperature (enzyme loading: 1200 µg $electrode^{-1}$/343 µg cm^{-2}).

Additional electrochemical notes, experiments, data and controls

Note S1: The OCV of a fuel cell is the highest voltage reached in the absence of any current flow. For an ideal case, the OCV will be given by the difference between the formal potentials of the fuel oxidized at the anode and the oxidant reduced at the cathode. In practice, however, each of these reactions proceed with a certain overpotential as a function of the employed (bio)catalyst and the experimental thermodynamic parameters. Therefore, the OCV reached by an enzymatic biofuel cell is also influenced by the overpotential required to drive the enzyme-catalysed reactions. Moreover, when a separate redox mediator species is used to shuttle electrons between the enzyme and electrode, associated losses in the OCV occur and the potential will now be determined by the difference between the formal potentials of the redox mediators involved in the electron transfer, in addition to any inherent overpotentials.

The use of redox mediators immobilized on the electrode surface (e.g. in the case of redox polymers) provides a pseudocapacitive component to the electrode assembly capable of storing charges in the form of oxidized/reduced redox centres due to enzymatic conversion in the presence of substrate. The established gradient in activity ratio implies a shift in the redox potential of the bioelectrode due to a self-charging process occurring at open circuit. In consequence, the experimentally recorded OCV (the voltage difference between the bioelectrodes) is effectively increased with respect to the apparent thermodynamic value defined by the midpoint potential of the polymer-bound redox mediator. For a more comprehensive discussion of this effect see ref. [5]

Table S1: Enzyme activities (H_2 oxidation), current densities of polymer/hydrogenase gas diffusion bioanodes and their power densities when incorporated into H_2/O_2 biofuel cells. All hydrogenases were immobilized on and wired to carbon cloth-based gas diffusion electrodes by means of the polymer double layer system P(GMA-BA-PEGMA)-vio//P(N_3MA-BA-GMA)-vio/hydrogenase.

hydrogenase	activity (H_2 Oxidation) [s^{-1}]	J_{max} [mA cm^{-2}]	P_{max} [mW cm^{-2}]	*enzyme loading* [nmol cm^{-2}]	I_{max}/*enzyme loading* [mA nmol^{-1}]	*total polymer loading* [mg cm^{-2}]	Ref.
*Dv*MF-[NiFe]	1000[6]	7.9	3.6 (at 0.7 V)	31.8	0.25	1.8	[2]
wild type *Dv*H-[NiFeSe]	4850[7]	5.3	-	27.0	0.20	1.8	[2]
wild type *Dv*H-[NiFeSe]	4850[7]	3.6	1.9 (at 0.7 V)	12.1	0.30	1.8	[2]
*Dv*H [NiFeSe] variant G491S	2416[8]	6.3	4.4 (at 0.7 V)	8.4	0.75	2.1	[8]
***Dd*HydAB [FeFe]**	**63 000[3]**	**14.1**	**5.4 (at 0.7 V)**	**39.8**	**0.36**	**2.1**	**this work**

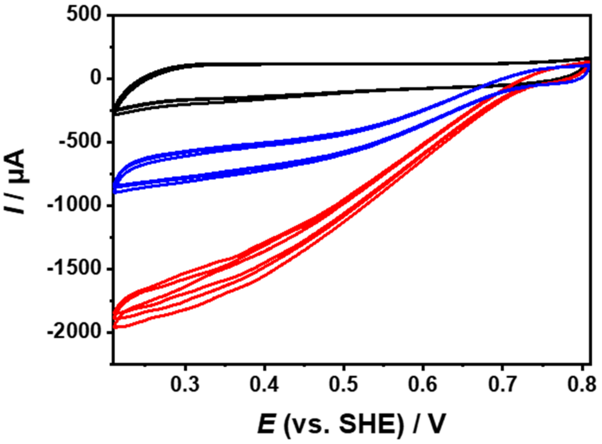

Figure S1: Multiple cyclic voltammograms of the Mv-BOx gas-diffusion biocathode under turnover conditions (red and blue traces, O_2) and under non-turnover conditions (black traces, Ar). Voltammograms under turnover conditions were measured before (red) and after the long term biofuel cell test (blue). Working electrolyte: 0.1M phosphate buffer (pH 7.4); scan rates for all voltammograms: 5 mV s^{-1}.

The biocathode showed absolute O_2 reduction currents of almost 2 mA (Figure S1, red curves), which is similar to the H2 oxidation currents observed for the best performing P(GMA-BA-PEGMA)-vio//P(N_3MA-BA-GMA)-vio/DdHydAB electrodes (cf. Figure 2a). Hence, to ensure anode limiting conditions, a bioanode with H2 oxidation currents slightly below the absolute currents of the biocathode was employed (Figure S2a, note that the bioanodes show considerable scatter in their absolute current values).

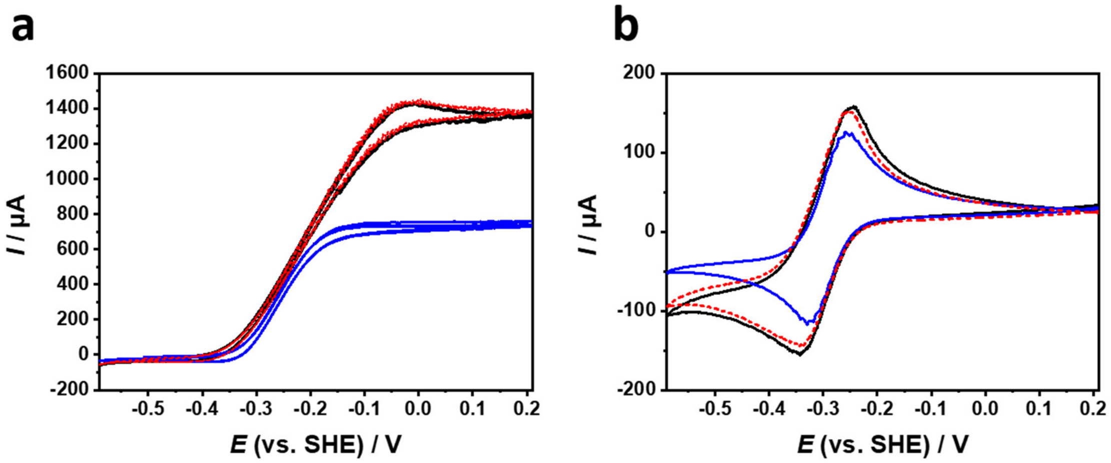

Figure S2: Cyclic voltammograms of a P(GMA-BA-PEGMA)-vio//P(N_3MA-BA-GMA)-vio/*Dd*HydAB gas-diffusion bioanodes under turnover (**a,** 100 % H_2) and non-turnover (**b,** 100 % Ar) conditions, measured before (black traces), after the biofuel cell test (dashed red traces) and after the long term stability test (blue). Working electrolyte: 0.1 M phosphate buffer (pH 7.4); *scan rate* = 5 mV s^{-1}; nominal enzyme loading: 39.8 nmol cm^{-2}; total polymer loading: 1.8 mg cm^{-2}.

The bioanode not only shows a decreased H_2 oxidation activity after the long-term experiment but also less pronounced non-turnover redox waves under 100% Ar, suggesting loss of parts of the polymer/enzyme film during the measurement (Figure S2b). The loss of the polymer layer is most likely due to the harsh operating conditions during the long-term stability evaluation (local pH shift due to proton generation from H_2 oxidation and continuous reduction of incoming O_2 to H_2O_2 at the redox polymer matrix, which can induce polymer degradation[9]).

References

[1] A. Ruff, J. Szczesny, S. Zacarias, I. A. C. Pereira, N. Plumeré, W. Schuhmann, *ACS Energy Lett.* **2017**, *2*, 964.

[2] J. Szczesny, N. Marković, F. Conzuelo, S. Zacarias, I. A. C. Pereira, W. Lubitz, N. Plumeré, W. Schuhmann, A. Ruff, *Nat. Commun.* **2018**, *9*, 4715.

[3] J. A. Birrell, K. Wrede, K. Pawlak, P. Rodriguez-Maciá, O. Rüdiger, E. J. Reijerse, W. Lubitz, *Isr. J. Chem.* **2016**, *56*, 852.

[4] a) N. Marković, F. Conzuelo, J. Szczesny, M. B. González García, D. Hernández Santos, A. Ruff, W. Schuhmann, *Electroanalysis* **2019**, *31*, 217; b) H.-q. Xia, K. So, Y. Kitazumi, O. Shirai, K. Nishikawa, Y. Higuchi, K. Kano, *J. Power Sources* **2016**, *335*, 105.

[5] a) S. Alsaoub, F. Conzuelo, S. Gounel, N. Mano, W. Schuhmann, A. Ruff, *ChemElectroChem* **2019**, *6*, 2080; b) F. Conzuelo, N. Marković, A. Ruff, W. Schuhmann, *Angew. Chem. Int. Ed. Engl.* **2018**, *57*, 13681.

[6] J. Aketagawa, K. Kobayashi, M. Ishimoto, *J. Biochem.* **1983**, *93*, 755.

[7] S. Zacarias, A. Temporão, M. d. Barrio, V. Fourmond, C. Léger, P. M. Matias, I. A. C. Pereira, *ACS Catal.* **2019**, *9*, 8509.

[8] A. Ruff, J. Szczesny, M. Vega, S. Zacarias, P. M. Matias, S. Gounel, N. Mano, I. A. C. Pereira, W. Schuhmann, *ChemSusChem* **2020**.

[9] H. Li, U. Münchberg, A. A. Oughli, D. Buesen, W. Lubitz, E. Freier, N. Plumeré, *Nat. Commun.* **2020**, 11, 920
